# Contactless Palmprint Recognition Using Binarized Statistical Image Features-Based Multiresolution Analysis

**DOI:** 10.3390/s22249814

**Published:** 2022-12-14

**Authors:** Nadia Amrouni, Amir Benzaoui, Rafik Bouaouina, Yacine Khaldi, Insaf Adjabi, Ouahiba Bouglimina

**Affiliations:** 1 LIST Laboratory, University of M’Hamed Bougara Boumerdes, Avenue of Independence, Boumerdes 35000, Algeria; 2 Electrical Engineering Department, University of Skikda, BP 26, El Hadaiek, Skikda 21000, Algeria; 3 PIMIS Laboratory, Electronics and Telecommunications Department, Université du 8 Mai 1945 Guelma, Guelma 24000, Algeria; 4 LIMPAF Laboratory, Department of Computer Science, University of Bouira, Bouira 10000, Algeria; 5 Higher School of Computer Science and Technology (ESTIN), Bejaia 06300, Algeria

**Keywords:** biometrics, palmprint recognition, wavelet analysis, multiresolution analysis, texture descriptors, binarized statistical image features

## Abstract

In recent years, palmprint recognition has gained increased interest and has been a focus of significant research as a trustworthy personal identification method. The performance of any palmprint recognition system mainly depends on the effectiveness of the utilized feature extraction approach. In this paper, we propose a three-step approach to address the challenging problem of contactless palmprint recognition: (1) a pre-processing, based on median filtering and contrast limited adaptive histogram equalization (CLAHE), is used to remove potential noise and equalize the images’ lighting; (2) a multiresolution analysis is applied to extract binarized statistical image features (BSIF) at several discrete wavelet transform (DWT) resolutions; (3) a classification stage is performed to categorize the extracted features into the corresponding class using a K-nearest neighbors (K-NN)-based classifier. The feature extraction strategy is the main contribution of this work; we used the multiresolution analysis to extract the pertinent information from several image resolutions as an alternative to the classical method based on multi-patch decomposition. The proposed approach was thoroughly assessed using two contactless palmprint databases: the Indian Institute of Technology—Delhi (IITD) and the Chinese Academy of Sciences Institute of Automatisation (CASIA). The results are impressive compared to the current state-of-the-art methods: the Rank-1 recognition rates are 98.77% and 98.10% for the IITD and CASIA databases, respectively.

## 1. Introduction

Biometric recognition methods are now the most often used for identifying or verifying people. Biometrics has supplanted traditional authentication techniques such as passwords or badges, which may be easily forgotten or lost [1]. In favor of biometric recognition systems, they cannot be stolen or forgotten since they are unique to each individual [2]. Biometric modalities can be classified into behavioral, chemical, and physical traits [3].

The palmprint as a biometric recognition modality has recently gained more popularity than other biometric characteristics; the palmprint modality is considered among the most effective tools in increasing the security of a person’s authentication [4,5]. Palmprint refers to the inside surface of the hand, which contains unique characteristics such as primary lines, wrinkles, ridges, and textures that even twins have different patterns [6]. Palmprint features remain stable and permanent during human life [7]. Biometric recognition based on palmprints is regarded as among the most practical and reliable authentication approaches compared to other physiological traits [8]. It has several advantages: the cheap cost, ease of access, acceptability by people, and higher distinctiveness [9].

Images of palmprints can be acquired in contact and non-contact (i.e., contactless) modes. In the context of a contact-based palmprint acquisition technique, the subject should place their hand in touch with the sensor attached to pegs to ensure that the hand is correctly positioned in order to take photos. In contrast, contactless acquisition is possible with commercial off-the-shelf cameras and under unconstrained conditions. The last mode provides several advantages over contact-based methods, such as increased user-friendliness and more confidentiality, and it does not provoke the hygiene risk [10].

The feature extraction stage is the most critical and delicate process in the biometric recognition system. If the algorithm used in this phase does not react appropriately, the system will automatically be flawed; this is why the feature extraction stage of the presented work piques our curiosity.

Local texture descriptors have gained significant research interest in the last few years. They describe pictures in small local patches and have been proven more efficient than global or geometric image descriptors; this is why we have chosen the local texture descriptor binarized statistical image features (BSIF) [11] in this research. In many computer vision applications, the BSIF descriptor has outperformed the performance of several homolog descriptors, such as the local binary patterns (LBP) [12], histograms of oriented gradients (HOG) [13], local phase quantization (LPQ) [14], or patterns of oriented edge magnitudes (POEM) [15]. Its principal consists of using the independent component analysis (ICA) [16] to train a collection of filters from real-world images.

The trained filters can be utilized to represent each pixel of the provided image as a binary string code by simply computing a convolution operation between its initial value and a learned filter. The binary code corresponding to the pixel may be considered a local descriptor of the picture intensity pattern in the pixel’s neighborhood. Finally, the histogram of pixel code values can be used to characterize texture features within picture sub-regions (i.e., multi-patch).

This study proposes a feature extraction approach for contactless palmprint recognition based on discrete wavelet transform (DWT) [17] multiresolution analysis and multi-scale BSIF description. The wavelet-based multiresolution analysis is a helpful tool that permits the analysis of the image and the extraction of pertinent information at multiple scales or resolutions. The BSIF descriptor is then applied to the original image in addition to the approximation coefficients (i.e., sub-images) extracted from each DWT level. It is well-known that the application of BSIF descriptor generates a feature vector in the form of a histogram representing the image.

As the BSIF descriptor is applied to the original image in addition to several DWT levels, i.e., each level will generate a corresponding histogram. In our approach, we have concatenated all histograms extracted from the same image (i.e., multi-level histograms) and generated a global histogram representing the image at several scales. So, we have performed a multiresolution image analysis and extracted the pertinent information from several resolutions.

For the classification stage, we have implemented and tested the performance of our approach with two classifiers, namely the K-nearest neighbors (K-NN) and centroid displacement-based K-nearest neighbors (CDNN). In addition, we conducted a deeper experimental analysis to find the best-performing parameters that present the higher recognition accuracy using two concurrent databases: the Indian Institute of Technology—Delhi (IITD) [18] and the Chinese Academy of Sciences Institute of Automatisation (CASIA) [19]. By comparing the obtained results against recently published state-of-the-art approaches, our method presents a competitive performance outperforming most of its contemporary approaches.

In addition, it has lower algorithmic complexity, i.e., lower computational cost than deep learning-based approaches, which take much time and require high-performance hardware usage.

In summary, the main contributions of our paper can be synthesized as follows:

- We proposed a feature extraction approach for contactless palmprint recognition based on DWT multiresolution analysis and multi-scale BSIF description.
- We substituted the classical approach of multi-block (i.e., multi-patch) decomposition with DWT multiresolution analysis.
- We conducted more profound experiments and analyses on the pre-processing, feature extraction, and classification stages by testing and considering several configurations to find the best-performing parameters that maximize the recognition rate.

The remaining paper is structured as follows: Section 2 includes a synopsis of the related work. Section 3 explains the proposed methodology and each technique used in this work. Section 4 displays our experimental results and a comparative study. The conclusion is introduced in Section 5.

## 2. Related Work

The following methodologies are the most often employed in the research for palmprint recognition: coding-based methods, texture-descriptor-based methods, and deep-learning-based methods.

### 2.1. Coding-Based Methods

These methods use filters to extract characteristics from palmprint images, which are subsequently encoded into digital codes. More precisely, they convert the responses of a bank of filters into bitwise codes and employ various distance metrics to compare and match palmprint images.

Kong and Zhang [20] proposed the competitive code, a method for palmprint identification based on a competitive coding scheme and angular matching. The authors applied the real part of the neurophysiology-based 2D Gabor filters to the palmprint to derive orientation information from the palmprint. In a proposed framework by Sun et al. [19], ordinal measures have been used to solve the representation problem. The authors introduced a different representation strategy called orthogonal line ordinal features that unifies several existing palmprint algorithms into the proposed framework.

The method produces a one-bit feature code by qualitatively comparing two elongated line-like image regions that are orthogonal. Tens of thousands of ordinal feature codes make up a palmprint pattern. Zhang et al. [21] analyzed the fragile bits phenomena in the palmprint’s binary orientation co-occurrence vector (BOCV) representation. If the value of a bit fluctuates in code maps made from many images of the same palmprint, that bit is said to be fragile. Next, they extended BOCV to E-BOCV by appropriately integrating information about fragile bits.

Fei et al. [22] tackled the problems of orientation feature extraction and effective matching of palmprint images. A new dual orientation code (DOC) scheme was developed to describe the palmprint’s orientation features. A good nonlinear angular match score was created to evaluate the similarity of DOCs. Xu et al. [23] proposed a new approach for palmprint authentication based on discriminative and reliable competitive coding and using a more precise dominant orientation representation of palmprint images. The authors suggested weighting the orientation data of a nearby region to enhance the robust and dominant discriminating orientation code’s correctness and stability.

### 2.2. Texture-Descriptors-Based Methods

These methods decompose the encoded palmprint image into several blocks, apply the texture descriptor to each block, and extract their histograms. Then, they concatenate the extracted histograms of each block to obtain the feature vector representing the palmprint image. Finally, they compare and match palmprint images using various distance metrics.

Motivated by the public desire for clean and non-intrusive biometric technologies, Michael et al. [24] proposed a touchless palmprint model for palmprint biometrics using a low-resolution web camera to acquire pictures. The authors used the skin-color thresholding approach to derive the region of interest (ROI). Then, a valley detection process was utilized to locate the valleys of the fingers as the proper points to find the palmprint area. The discriminative palmprint features are obtained by applying the LBP texture descriptor to the directional gradient responses of the palmprint.

Morales et al. [25] analyzed the difficulties of two palmprint techniques used for contactless biometric authentication, namely the orthogonal line ordinal features (OLOF) and scale-invariant feature transform (SIFT) features. The authors evaluated their performance in the presence of large-scale, rotation, occlusion, and translation fluctuations.

Hammami et al. [26] focused on areas of the image containing the most discriminating characteristics for identification. The authors located, extracted, and preprocessed the ROI. Then, the ROI was divided into sub-regions before applying the LBP texture descriptor for feature extraction. Finally, LBP histograms are concatenated in the form of a global histogram. Wu et al. [27] suggested a SIFT-based method to extract and match features for contactless palmprint identification. They utilized two steps in the matching stage to eliminate the mismatched SIFT points. In the first stage, SIFT has been coupled with RANSAC (i.e., random sample consensus) method, and in the second stage, LPDs (i.e., local palmprint descriptors) were used.

Another contactless palmprint recognition approach has been proposed by Luo et al. [28] that is based on SIFT feature extraction. First, palmprint images are preprocessed with an isotopic filter before detecting and matching SIFT points. Next, mismatched points that do not satisfy the topological relations are eliminated using the iterative-RANSAC (I-RANSAC) algorithm. LPDs are retrieved for SIFT to eliminate the mismatched data. Wu et al. [29] proposed a variation of the LBP texture descriptor in the local line-geometry space for palmprint recognition called local line directional patterns (LLDP). By examining directional line data, LLDP encodes a neighborhood’s structure. Consequently, using the modified finite random transform (MFRAT) or Gabor filters, the line responses in the neighborhood are calculated in 12 dissimilar directions.

### 2.3. Deep Learning-Based Methods

These approaches often use convolutional neural networks (CNN). CNNs are composed of convolutional, pooling, and fully connected layers that can simultaneously be trained and utilized for feature extraction and classification.

The authors in [30] proposed a heuristic palmprint identification approach that extracts three types of palmprint features from the image. First, they extracted the texture, gradient, and direction features to be encoded in triple-type feature codes. Then, they created the triple feature descriptors for palmprint representation using the block-wise histograms of the triple-type feature codes. The similarity between two matched triple-type feature descriptor palmprint images was then determined using a weighted matching-score level fusion.

In a recent study by Fei et al. [5], the authors summarized the feature extraction and recognition of different palmprint images using a unified framework to categorize palmprints into four categories. Then, they analyzed the motivations and theories of the representative extraction and matching methods. Among these methods, the authors analyzed the effectiveness of the deep-learning-based models on palmprint recognition.

Zhao and Zhang [31] suggested a new deep discriminative representation (DDR) strategy. DDR is a palmprint recognition technique based on learning the discriminative deep convolutional networks (DDCN). DDCNs are trained using constrained data to derive deep discriminative patterns (global and local) from palmprint images. They utilized CRC (i.e., collaboration representation based on a classifier) for the classification stage.

Table 1 recapitulates the works synthesized in this section by approaches, databases employed, and experimental protocols. Motivated by the successes of the second class, “Texture-descriptors-based methods”, we proposed in this work a practical approach called “binarized statistical image features-based multiresolution analysis” to handle the problem of palmprint recognition. Furthermore, most methods of the second class are based on image multi-bloc decomposition to extract the pertinent information from several levels. We proposed an alternative solution to the classical multi-bloc image decomposition strategy in this work. Our solution adopts the multiresolution analysis with the help of the DWT, which effectively extracts pertinent information from several levels.

## 3. Proposed Approach

This paper proposes a three-step approach to address the challenging problem of contactless palmprint recognition: (1) a pre-processing, based on median filtering and contrast limited adaptive histogram equalization (CLAHE), is used to remove potential noises and equalize the images’ lighting; (2) a multiresolution analysis is applied to extract BSIF features at several DWT scales; (3) a classification stage is performed to categorize the extracted features into the corresponding class using the K-NN or CDNN classifiers. This section details the fundamental concepts of these approaches. We used the pre-processing phase before the feature extraction and classification processes, which significantly influences the performance and robustness of the recognition rate. We used median filtering and CLAHE for the pre-processing stage.

### 3.1. Pre-Processing

We used the pre-processing phase before the feature extraction and classification processes, which significantly influences the performance and robustness of the recognition rate. We used median filtering and CLAHE for the pre-processing stage.

Median filtering is a simple implementation of a nonlinear filter that is essential in image processing since it is widely recognized for preserving picture borders during noise reduction, particularly “Salt & Pepper” noise [32]. The median filter replaces each pixel’s gray level with the median value of the gray levels in the pixels’ neighborhood [33].

CLAHE is a line contrast enhancement pre-processing tool. CLAHE involves conducting histogram equalization on non-overlapping picture sub-areas and applying interpolation to repair irregularities across boundaries [34].

The technique includes the following steps: (1) CLAHE divides the input picture into non-overlapping sub-blocks, (2) it generates the histograms for each sub-block, (3) the clip limit notion is then used; the clip limit is a multiple of the histogram’s average content (each histogram is redistributed to ensure its height does not exceed a predetermined “clip limit”), (4) the cumulative histogram is calculated to perform the equalization, and (5) finally, bilinear interpolation is used between the blocks to eliminate block distortions [35].

### 3.2. Feature Extraction

In this section, we first describe the theoretical fundaments of the methods used in this study and then present the working procedure of our proposed feature extraction approach.

#### 3.2.1. Theoretical Principle of the Employed Methods

A. Discrete Wavelet Transform (DWT)

Wavelets have been used in many applications, including feature extraction, compression, denoising, and contour detection. DWT divides a given signal into several frames, each of which is a time series of coefficients characterizing the signal’s temporal evolution in the appropriate frequency band [36,37]. The DWT works with many mother wavelets, including Haar, Daubechies, Coiflets, symlet, etc. [36,38].

The theoretical framework to apply a DWT on a 2D image is expressed mathematically with Equations (1) to (7) and explained as follows:

Suppose ψ(t) is a function of the mother wavelet. The wavelet function family ψ(s,p)(t) can be obtained as [39,40]:

(1) $$\psi \left(s,p\right)\left(t\right)=\frac{1}{\sqrt{s}}\;\Psi \left(\frac{t-p}{s}\right)$$

where s is the scale parameter, t is an instance, and p is the position parameter.

Let f(x,y) be an image of a size M×N. The 2D-DWT is expressed as follows:

(2) $$\;\;\;\;\;\;\;\;\;W_{\varphi }\left(j_{0},m,n\right)=\frac{1}{\sqrt{MN}}\;\overset{M-1}{\underset{x=0}{\sum }}\overset{N-1}{\underset{y=0}{\sum }}f\left(x,y\right)\varphi_{j_{0},m,n}\left(x,y\right)\;\;\;\;\;\;\;\;\;\;\;\;\;\;\;$$

(3) $$W_{\psi }^{i}\left(j,m,n\right)=\frac{1}{\sqrt{MN}}\;\overset{M-1}{\underset{x=0}{\sum }}\overset{N-1}{\underset{y=0}{\sum }}f\left(x,y\right)\psi_{j,m,n}\left(x,y\right)$$

where i={horizontal, vertical, diagonal}, $W_{\varphi }\left(j_{0},m,n\right)$ are the coefficients that define an approximation of f(x,y) at a scale $j_{0}$, $W_{\psi }^{i}\left(j,m,n\right)$ are the coefficients that add horizontal, vertical, and diagonal details for a scale $j\geq j_{0}$, φ defines the scaling function, and ψ defines the wavelet function.

**Figure 1:**
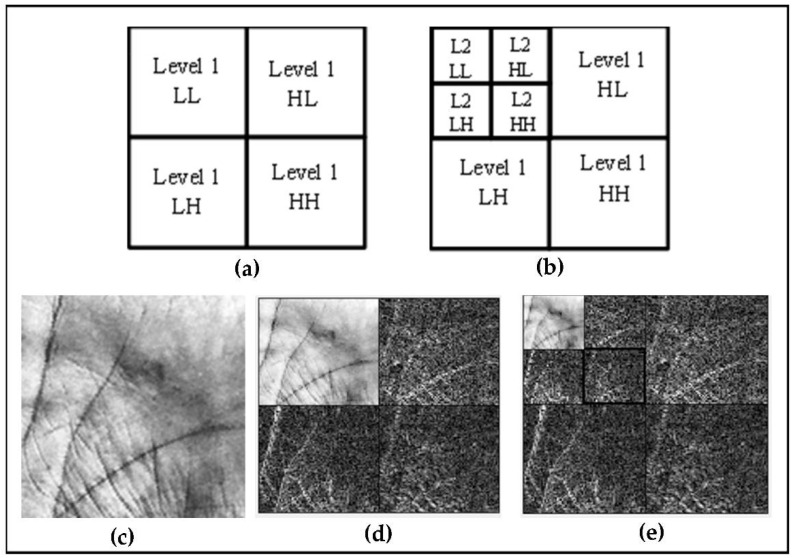
The 2D wavelet decomposition. (**a**) Concept of one-level components. (**b**) Concept of two-level components. (**c**) Original palmprint image from IITD database. (**d**) The 2D-DWT at first level. (**e**) The 2D-DWT at second level.

In a picture, several resolutions are represented by repeating cycles of scaling (low pass) and wavelet transform (high pass), as depicted in Figure 1. The scaling catches the image’s low-frequency information, while the wavelet collects the image’s high-frequency information. At each cycle of the wavelet transform, a low-resolution picture and a fine details image, each half the size of the original image, are generated. As the information in fine details images is frequently limited, the lower-resolution version captures most of the information in the original image, resulting in high image representation efficiency [41,42].

The 2D wavelet divides a picture into four sub-band images: Low-Low (LL), Low-High (LH), High-Low (HL), and High-High (HH), as displayed in Figure 1. Low frequency indicates approximation coefficients, while high frequency represents detail coefficients (horizontal, vertical, and diagonal). Mathematically, this procedure can be presented as follows.

Let φ define the scaling function, and ψ define the wavelet function. As indicated in Equations (4)–(7), the DWT produces four quarter-sized pictures at each level of decomposition: overview image φ(x, y), horizontal details image $\psi^{H}\left(x,\;y\right)$, vertical details image $\psi^{V}\left(x,\;y\right)$, and diagonal details image $\psi^{D}\left(x,\;y\right)$ [39,41,42].

(4) φ(x,y)=φ(x)φ(y) →LL

(5) $$\;\psi^{H}\left(x,y\right)=\varphi \left(x\right)\psi \left(y\right)\to HL$$

(6) $$\;\psi^{V}\left(x,y\right)=\psi \left(x\right)\varphi \left(y\right)\to LH$$

(7) $$\psi^{D}\left(x,y\right)=\psi \left(x\right)\psi \left(y\right)\to HH$$

In the following example, DWT frequency components for two distinct scale values are generated, and the acquired components’ low-frequency sections (LL, LLLL) are used in processes.

B. Binarized Statistical Image Features (BSIF)

The binarized statistical image features (BSIF) descriptor introduced by Kannala and Rahtu [11] was used for texture description and classification, which is based on local binary patterns (LBP) [12] and local phase quantization (LPQ) [14]. The fundamental concept behind this descriptor is to employ learned filters with independent component analysis (ICA) [16] rather than handcrafted filters to calculate a binary code string for each pixel in an image, which can then be used to maintain an efficient histogram representation.

The BSIF approach generates a binary code string from image pixels, with the code value serving as a local descriptor of the picture intensity model in the pixel’s neighbors. Histograms of pixel code values can also quantify texture qualities within picture subspaces.

By binarizing the response of a linear filter with a zero threshold, the value of each bit in a binary code string is determined [43]. Each bit is associated with a unique filter, and the number of filters used is determined by the appropriate length of the bit string. The filters are trained using a training set of natural image patches. The learning process is carried out by maximizing the statistical independence of the filter responses [44,45].

For a given image patch X of size l×l pixels and a linear filter $W_{i}$ of the same size, the $s_{i\;}$ filter response is determined as follows [11]:

(8) $$s_{i}=\underset{u,v}{\sum }W_{i}\left(u,v\right)\;X\left(u,v\right)=w_{i}^{T}x$$

where $s_{i}$ denotes the filter response, $W_{i}$ denotes a linear filter, (u,v) denotes the spatial coordinates, i indicates the number of the filter, and the vectors w and x contain the pixels of $W_{i}$ and X, respectively.

The binarized feature $b_{i}$ is produced by:

(9) $$b_{i}=\{\begin{array}{c} 1\;\;\;\;\;\;\;if\;s_{i}>0\\ 0\;\;\;\;otherwise \end{array}$$

Given n linear filters $W_{i}$, which can be stacked to a matrix W of size $n\times l^{2}$, and computing all responses at once, i.e., *s* =Wx, the bit string b is obtained by binarizing each element $s_{i}$ of s as described above.

Lastly, the BSIF features are obtained by treating each individual pixel as a combination of binary values determined from the n number of linear filters. BSIF encoded features β are obtained as follows:

(10) $$\beta =\overset{n-1}{\underset{i=0}{\sum }}b_{i}2^{i}$$

**Figure 2:**
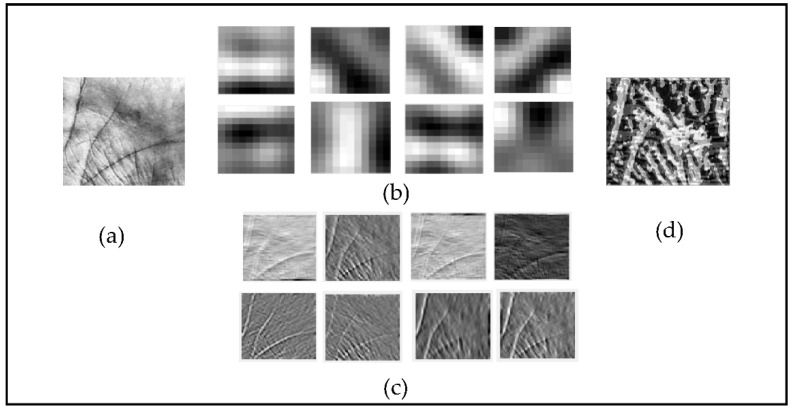
(**a**) Palmprint image, (**b**) pre-learned filter with a size of l×l=11×11 and bit string length of n=8, (**c**) BSIF features, and (**d**) final BSIF encoded features.

**Figure 3:**
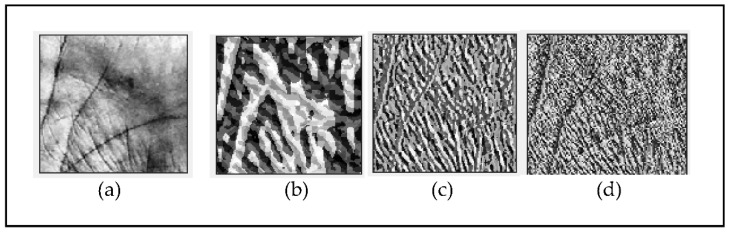
Example of BSIF conversion results for (**a**) original image, (**b**) l×l=17×17, n=8, (**c**) l×l=9×9, n=12, and (**d**) l×l=3×3, n=8.

Figure 2 depicts the entire BSIF extraction technique using an 11×11 BSIF filter with a bit string length of 8. Figure 2a illustrates the input ROI of a palmprint image, Figure 2b depicts pre-learned filters with a dimension of 11×11 and a bit string length of 8, Figure 2c represents the BSIF features derived by convolution of ROI images with BSIF filters, and the completed BSIF encoded features are shown in Figure 2d. Figure 3 depicts several BSIF findings with varying filter sizes and bit string lengths.

The filters $W_{i}$ are learned by improving the statistical independence of $s_{i}$ employing ICA. The BSIF descriptor has two parameters: the filter size l and the bit string length n [43]. The initial filters $W_{i}$ presented by [11,46] were learned by randomly sampling 50,000 picture patterns from 13 distinct natural photographs.

#### 3.2.2. Proposed Feature Extraction Approach—Multiresolution Analysis

**Figure 4:**
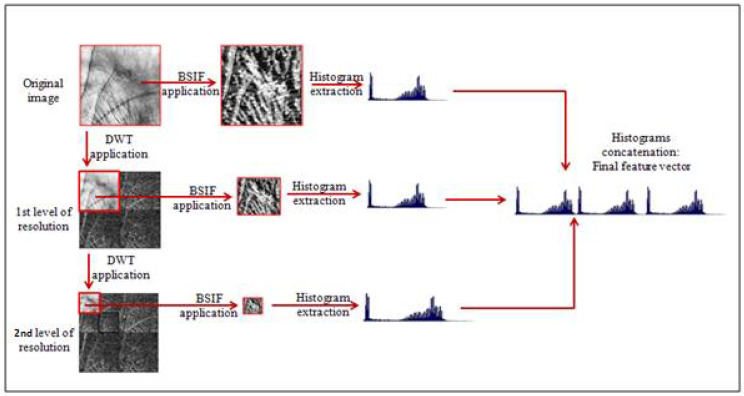
Graphical flowchart of the proposed feature extraction approach.

The feature extraction stage, which is the main contribution of this study, is based on a multiresolution analysis of the entered palmprint image. As shown in the graphical flowchart of Figure 4, the concept consists firstly of applying the BSIF descriptor on the original image and extracting its corresponding histogram, which defines the feature vector of the original image. Secondly, we apply the DWT decomposition on the original image, which will decompose this image into four sub-band images: Low-Low (LL), Low-High (LH), High-Low (HL), and High-High (HH) images, as explained in Section 3.2.1.

This decomposition represents the first level (or resolution) of the DWT decomposition. It is well-known that the pertinent information is concentrated in the approximations coefficients (i.e., LL sub-band), while the detail coefficients, which are high frequencies, generally contain not useful information (mostly noises or contours). In addition, the generated LL image will replace and compress the original image and permit access to more precise representations of the original image.

For the first level of decomposition, we also apply the BSIF descriptor on the L1-LL (i.e., Level 1 of Low-Low) sub-band and generate the corresponding histogram. Similarly, the process of DWT decomposition will be applied on the L1-LL sub-band to obtain the L2-LL (i.e., Level 2 of Low-Low) representation containing more useful and compressed information, i.e., the approximations coefficients of resolution 2.

This operation is followed by a BSIF application to generate the histogram of the second resolution. The process of DWT decomposition is repeated for N decomposition levels depending on the size of the image. Finally, the histograms generated from each level are merged to create a global feature vector representing the palmprint at several levels. In other words, we have applied a multiresolution analysis on the palmprint image, as shown in Figure 4.

### 3.3. Classification

For the classification stage, we have implemented, tested, and compared the performance of two classifiers, namely K-NN and CDNN.

#### 3.3.1. K-Nearest Neighbors (K-NN) Classifier

The K-nearest neighbors (K-NN) classifier is an easy-to-understand and simple-to-implement classification approach. The K-NN algorithm was used for classification, pattern recognition, and image processing. This method has three main components: a set of labeled items, a distance function that assesses the difference or similarity between two items, and the amount of K, i.e., the number of nearest neighbors. To categorize an unlabeled object, the distance between it and the labeled items is first calculated to determine its K nearest neighbors, and then the item is classified based on the majority class of its K nearest neighbors [47,48]. We have employed two distance metrics in this study: *Euclidean* and *City Block*.

The Euclidean distance D(A,B) between two samples A and *B* is formally defined as [49]:

(11) $$D\left(A,B\right)=\sqrt{\overset{N}{\underset{i=1}{\sum }}{\left(a_{i}-b_{i}\right)}^{2}}$$

The City Block distance is calculated as follows:

(12) $$D\left(A,B\right)=\overset{N}{\underset{i=1}{\sum }}\left(a_{i}-b_{i}\right)$$

where D(A,B) is the distance between test sample A and specified training sample B of features (1,2,3,…,N), $a_{i}$ are the features of the test sample of A, $b_{i}$ are the features of the specified training sample of B, and N is the total number of features.

#### 3.3.2. Centroid Displacement-Based K-Nearest Neighbor (CDNN) Classifier

We utilized CDNN for classification, which is noise and class distribution adaptable. CDNN is a modified K-NN method published by Nguyen et al. [50] that addresses the issue of the majority vote in the K-NN algorithm: if the distance between the test sample and its neighbors varies greatly, the closer neighbors more consistently predict the class label. The CDNN algorithm works on a basic principle: once the K-NN of x (test sample) list $D_{x}^{k}$ is obtained, the nearest neighbors are organized into sets with the same class designation,$\;S_{x}^{j}=\left\{\left(x_{t},c_{t}\right)\in D_{x}^{k}\;|\;c_{t}=c^{j},\;c^{j}\in C\right\}$, where C is the class label of x, $x_{t}$ is the feature vector in instance *t*, $x_{t}=(x_{t1},x_{t2},\ldots ,x_{tN}$), and $c_{t}$ is the class label of $x_{t}$.

The centroid of each set and its displacement if the test sample x is added to the test are computed as follows.

The centroid of $S_{x}^{j}$ is calculated as:

(13) $$p_{x}^{j}=\frac{\sum_{\forall x_{t}\in \;S_{x}^{j}}x_{t}}{\left|S_{x}^{j}\right|}$$

where $p_{x}^{j}$ is the centroid of $S_{x}^{j}$,$\;S_{x}^{j}=\left\{\left(x_{t},c_{t}\right)\in D_{x}^{k}\;|\;c_{t}=c^{j},\;c^{j}\in C\right\}$, C is the class label of x, $x_{t}$ is the feature vector in instance *t*, $x_{t}=(x_{t1},x_{t2},\ldots ,x_{tN}$), $c_{t}$ is the class label of $x_{t}$, and $D_{x}^{k}$ is the list of K-nearest neighbors to x.

The new centroid if x is inserted into $S_{x}^{j}$ is calculated as:

(14) $$q_{x}^{j}=\frac{(\sum_{\forall x_{t}\in \;S_{x}^{j}}x_{t})+x}{\left|S_{x}^{j}\right|+1}$$

where $q_{x}^{j}$ is a new centroid if x is inserted into $S_{x}^{j}$, $\;S_{x}^{j}=\left\{\left(x_{t},c_{t}\right)\in D_{x}^{k}\;|\;c_{t}=c^{j},\;c^{j}\in C\right\}$, C is the class label of x, $x_{t}$ is the feature vector in instance *t*, $x_{t}=(x_{t1},x_{t2},\ldots ,x_{tN}$), $c_{t}$ is the class label of $x_{t}$, and $D_{x}^{k}$ is the list of K-nearest neighbors to x.

The centroid displacement is calculated as:

(15) $$disp_{x}^{j}=\sqrt{{\left(p_{x}^{j}-q_{x}^{j}\right)}^{2}}$$

where $disp_{x}^{j}$ is the centroid displacement,$\;p_{x}^{j}$ is the centroid of $S_{x}^{j}$,$\;q_{x}^{j}$ is a new centroid if x is inserted into $S_{x}^{j}$.

Finally, the test sample is assigned to the assortment with the lowest centroid displacement.

## 4. Experimental Analysis

The proposed method was assessed using the IITD touchless palmprint (version 1.0) [18] and CASIA palmprint [19] datasets. This section discusses the specifications of each employed dataset and its assessment protocol. Moreover, we examine the results achieved from applying our suggested method and compare the Rank-1 recognition rates with other recent state-of-the-art approaches.

More precisely, we have examined the results achieved from applying our suggested method and compared the Rank-1 recognition rates with other recently published state-of-the-art methods: five coding-based methods including Compcode [20], OrdinalCode [19], E-BOCV [21], DOC [22], and DRCC [23]; six texture-based methods, including DGLBP [24], SIFT_OLOF [25], LBP [26], SIFT_IRANSAC_OLOF [27], LLDP_MFRAT [28], LLDP_Gabor [28], and TFD [29]; in addition to three deep learning-based methods, including FEM [30], AlexNet [5], VGG-16 [5], Inception-V3 [5], ResNet-50 [5], and DDR [31].

### 4.1. Datasets

#### 4.1.1. IITD Touchless Palmprint Database (Version 1.0)

**Figure 5:**
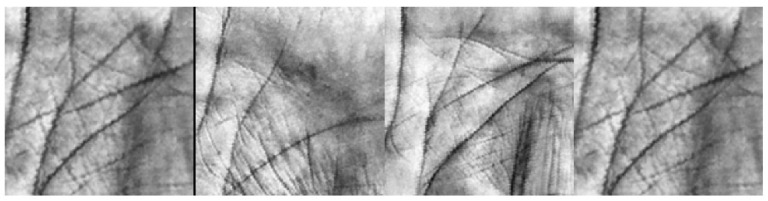
Sample images from the IITD database.

The IITD (i.e., Indian Institute of Technology—Delhi) touchless palmprint database [18] was created in the biometric research laboratory from January 2006 to July 2007. The database was collected from 230 users with several images from both hands of each user. The age of participants ranges from 12 to 57 years. The database employed user-pegs to reduce the hand-pose and image scale variations. The resolution of the original images is 800 × 600 pixels and 150 × 150 pixels for cropped and normalized palmprint images. Figure 5 shows some sample images from the IITD database.

#### 4.1.2. CASIA Palmprint Database

**Figure 6:**
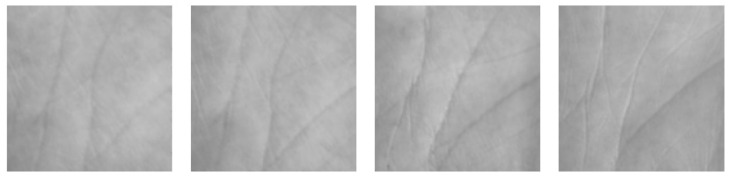
Sample images from the CASIA palmprint database.

CASIA palmprint database [19] was released by the Chinese Academy of Sciences Institute of Automatisation. The CASIA database contains 5502 palmprint images acquired from 312 persons who furnished 8 images for both right and left hands. All palmprint images were saved in 8 gray-level JPEG files. The sensor used for capturing the palm images provides uniform lighting but no pegs to reduce the postures and positions of palms. Some image examples are depicted in Figure 6.

### 4.2. Setups

In this study, all our experiments are conducted using the identification mode. Biometric identification attempts to identify the label of a query sample by comparing its feature vector to a collection of labeled samples (i.e., feature vectors) stored in a referential database. As reported in many recently published state-of-the-art papers, the evaluation protocol used in the identification experiments consists of selecting the first N images for each person to create the gallery set. The remaining examples of each person are used as query samples. Then, the Rank-1 identification rate is calculated to measure the performance of our approach and make a comparison against the related approaches.

The identification rate is calculated using the following formula:

(16) $$Identification\_Rate=\frac{Number\;of\;correct\;predictions}{Total\;number\;of\;testing\;samples}\times 100$$

It is well-known that 230 persons have participated in the formation of the IITD database using the left and right hands, i.e., the database includes 460 classes because each hand is considered an independent class. As the number of images per class varies between 5 and 6 images, the N value considered in this experiment is equal to 3, i.e., the first 3 images are used in training, and the remaining images (2–3) are used in testing.

Similarly, the CASIA dataset was generated with the participation of 312 persons that have used both hands, i.e., the database holds 624 classes because each subject has 8 left images and 8 right images. The number of images per person in this database is constant and equal to 8. The N value considered in this experiment is equal to 4, i.e., the first 4 images of each subject are used for training, and the remaining 4 images are used for testing.

### 4.3. Experiment #1 (Effects of the BSIF Parameters)

In the first experiment, we evaluated the suggested approach by experimenting with different BSIF settings to find the optimum configuration—that is, the one that produces the most outstanding recognition accuracy. The BSIF operator depends on two parameters: bit string length n and filter kernel size l×l, as described in Section 3.2.1. In this experiment, we have not applied any pre-processing, the BSIF descriptor was applied on the original image, i.e., without any image decomposition or application of DWT multiresolution analysis, and the classical K-NN with the Euclidean distance was used as a classifier. Table 2 and Table 3 show the detailed results of this first experiment on both employed databases. The highest results are marked in bold.

Table 2 and Table 3 show that the recognition rates increase with the augmentation in the size of filter kernel l×l or with the value of the bit string n. In addition, the configuration l×l=17×17 and n=12 is the best performing; the best-achieved recognition rates using this configuration are 96.35% and 95.49% for the CASIA and IITD, respectively.

### 4.4. Experiment #2 (Effects of the Multiresolution Analysis)

The feature extractor BSIF was applied only to the original image without any image decomposition or multiresolution analysis in the previous experiment. This second experiment aims to check if exploiting multiresolution information improves recognition performance. We try to answer the previous question by testing and assessing the recognition performance at different DWT decomposition levels. So, several levels of the multiresolution analysis were tested and compared in this experiment.

**Figure 7:**
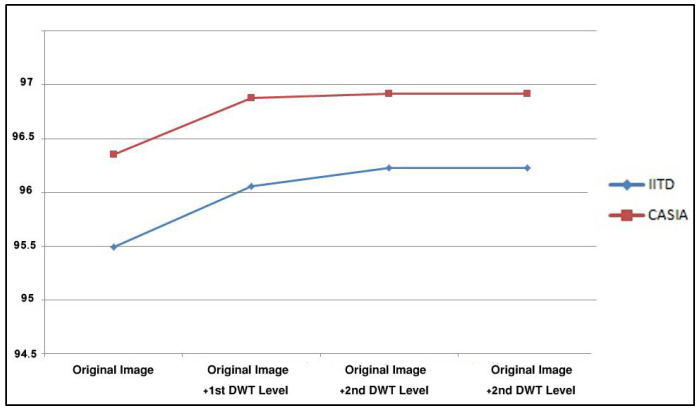
Recognition rates using several DWT decompositions applied to the CASIA and IITD databases.

In addition, the BSIF configuration considered in this experiment is a bit string length n=12 bits and a filter kernel size l×l=17×17 pixels (the best-performing configuration found in the previous experiment), the Haar wavelet is the DWT family considered in the experiment, no image pre-processing, and the classical K-NN with the Euclidean distance was used as a classifier. Figure 7 shows the detailed results of this experiment on both used databases.

Figure 7 shows that going deeper with DWT decomposition permits higher results until converging at level 2. We can conclude that DWT decomposition has a prominent role in extracting useful information permitting to improve recognition accuracy. We consider level 2 as the best-performing configuration because it achieves the best recognition rates for both databases, and its algorithmic complexity is minimal compared to the 3rd level (due to the less computation). Compared to the previous experiments, the recognition rates were improved from 95.49% to 96.23% and from 96.35% to 96.92% on IITD and CASIA databases, respectively.

### 4.5. Experiment #3 (Effects of the Wavelet Family)

Starting from the optimal parameters determined in the previous experiments, i.e., l×l=17×17, n=12, and 2nd DWT level, we assessed in this 3rd experiment the performance of recognition by testing and comparing several wavelet families. The most famous wavelet families are Haar, Daubechies (db), Biorthogonal (bior), Coiflets (coif), Symlets (sym), Fejer-Korovkin filters (fk), Reverse Biorthogonal (rbio), and Discrete Meyer. For more details on wavelet families, see [51]. Table 4 and Table 5 show the detailed results of this third experiment on both tested databases. Note that this experiment was applied using the classical K-NN with the Euclidean distance as a classifier and without any image pre-processing.

We can observe from Table 4 that Coiflets wavelet of types coif2 and coif4, as well as Daubechies wavelet type db4, achieve the best performance with a recognition rate = 96.39%, and the recognition rate was improved from 96.23% to 96.39% on IITD database. On the other hand, it can be observed from Table 5 that the Reverse Biorthogonal wavelet is the best performing, with a recognition rate of 97.12% and an improvement from 96.92% to 97.12% on the CASIA database. For the following experiments, we consider the wavelet family Coiflets type coif4 the optimal parameter as it presents a good compromise between both databases regarding the recognition rate.

### 4.6. Experiment #4 (Effects of the Pre-Processing)

The objective of this experiment is to show if image pre-processing can influence or increase the performance of our palmprint identification system. It is well-known in the image processing field that pre-processing covers two types of operations: filtering (i.e., eliminating noise or irrelevant information) and restoration (i.e., enhancing or equilibrating the image’s lighting). We applied median filtering before the feature extraction phase in the first attempt; median filtering is considered among the best image filtering operators, eliminating impulsion noises and preserving the image’s content. In the second attempt, we applied CLAHE to improve and restore the lighting of images (see Section 3.1 for more details about CLAHE pre-processing).

The identification results are presented in Table 6, and the best parameters found in the previous experiments are taken into consideration. We can observe from Table 6 that using median filtering as pre-processing harms the identification rate; this later decreased from 96.39% to 95.99% with the IITD database and from 97.12% to 96.60% with the CASIA database. So, applying an operation of filtering based on median filtering to our system is not suitable.

Table 6 also recorded the results of applying the CLAHE pre-processing to the IITD and CASIA databases.). With CLAHE, the recognition rates have been improved from 96.39% to 97.13% and from 97.12% to 97.21% with IITD and CASIA databases, respectively. In contrast to median filtering, CLAHE pre-processing positively impacts the identification performance.

### 4.7. Experiment #5 (Effects of Classification)

In the last experiment of this work, we tried to adjust and assess the parameters of classification. More precisely, we have considered two classifiers: K-NN and CDNN (see Section 3.3 for more details about these classifiers). In addition, it is well-known that K-NN should use a distance measure. For this reason, we have tested and considered two distance measures: Euclidean and City Block (explained in Section 3.3). Finally, choosing the K-value is essential as it represents an important element in finding the majority class from the nearest neighbors. For this reason, several K-values have been tested, considered, and compared.

In Table 7, we recorded the results of this experiment by varying the classifier, the distance measure, and the K-value for both databases, in addition to using the best-performing parameters found in the previous experiments.

From Table 7, it can be observed that augmenting the K-value causes a decrease in the recognition rates; the K=1 is the best choice for both classifiers and with both distances. In addition, the classification results of the K-NN surpass the results of the CDNN with K>1, but both classifiers have the same result with k=1, which is considered the best. Finally, the City Block distance performs well than the Euclidean distance with both databases. As a recapitulation, adjusting the classification parameters has improved the recognition rates from 97.13% to 98.77% and from 97.21% to 98.10% with IITD and CASIA databases, respectively.

### 4.8. Comparison

Table 8 presents the final classification accuracies of the IITD and CASIA datasets using our proposed BSIF + DWT approach and a comparison study to recently published approaches. The same experimental protocol was used for all tested techniques for a fair comparison. We can notice that our approach outperforms existing deep learning-based methods (e.g., FEM [30] and ResNet-50 [5]), texture-based methods (e.g., SIFT_IRANSAC_OLOF [27]), and other approaches. Our approach has the highest accuracy, with 98.77% and 98.10%, respectively, on the IITD and CASIA palmprint datasets, except for the work of Zhao and Zhang [31], named DDR, which has achieved 99.41% for the CASIA palmprint dataset.

The best results obtained using the BSIF-based multiresolution analysis on both IITD and CASIA datasets could be explained by using the powerful feature extractor BSIF on various DWT decomposition levels and adjusting the classification parameters.

In summary, the superiority of our approach is justified by the following points:

- The texture extractor analyses the picture pixel by pixel, i.e., we consider the advantages of local information.
- The picture is analyzed at several levels, i.e., we exploit multi-level information.
- The extracted occurrences from each level are collected in a histogram, i.e., we operate with global information.

## 5. Conclusions

Palmprint-based biometric recognition is a highly challenging issue that we attempted to solve by introducing a novel feature extraction strategy called *multiresolution analysis*, which is an alternative to the classical multi-patch decomposition strategy. More precisely, we have first applied the DWT on the original palmprint image, which permits the generation of several image representations (i.e., sub-bands) with different resolutions. Then, we applied the local texture descriptor BSIF on the original image as well as the generated image sub-bands of low-low resolutions. Finally, the extracted histograms from each level are merged to form the final feature vector, which represents the entered image at several resolutions.

We validated the performance of the proposed feature extraction approach by conducting more profound experiments using the recognition rate as a statistical measure as well as the IITD and CASIA palmprint databases. The best Rank-1 recognition rates achieved using our approach are 98.77% and 98.10% for the IITD and CASIA databases, respectively; these results are very competitive and surpass the performance of many recently published state-of-the-art approaches.

In the future, we intend to (1) implement the multiresolution analysis with a deep learning model as an alternative to the local texture descriptor BSIF and (2) improve the quality of training and render it more semantic by using the deep unsupervised active learning strategy.

## Figures and Tables

**Table 1:** Comprehensive summary of related work studies.

Approach	Publication	Method	Employed Dataset			Evaluation Protocol
			Name	#Sub.	#img.	
Coding-based methods	Kong and Zhang [20]	CompCode	IITD	230	2600	4 img/sub randomly selected Train and remaining Test
			CASIA	312	5500	
	Sun et al. [19]	OrdinalCode	IITD	230	2601	4/5 img/sub Train and remaining Test
			CASIA	312	5502	
	Zhang et al. [21]	E-BOCV	IITD	230	2601	2 img/sub Train and remaining Test
			CASIA	312	5502	3 img/sub Train and remaining Test
	Fei et al. [22]	DOC	IITD	230	2601	4/5 img/sub Train and remaining Test
			CASIA	312	5502	
	Xu et al. [23]	DRCC	IITD	230	2600	4 img/sub Train and remaining Test
			PolyU II	193	7752	
			MPolyU	250	6000	
Texture-based methods	Michael et al. [24]	DGLBP	CASIA	312	5500	4 img/sub Train and remaining Test
			IITD	230	2601	
	Morales et al. [25]	SIFT_OLOF	CASIA	312	5500	4 img/sub Train and 4 img/sub remaining Test
			IITD	230	2601	
	Hammami et al. [26]	LBP	CASIA	282	5412	4 img/sub Train and 4 img/sub remaining Test
			PolyU	193	7752	
	Wu et al. [27]	SIFT_IRANSAC_OLOF	CASIA	312	5500	4 img/sub Train and 4 img/sub remaining Test
			IITD	230	2601	
	Luo et al. [28]	LLDP_MFRAT	CASIA	312	5500	4 img/sub Train and 4 img/sub remaining Test
			IITD	230	2601	
		LLDP_Gabor	CASIA	312	5500	
			IITD	230	2601	
	Wu et al. [29]	TFD	CASIA	312	5502	4 img/sub Train and remaining Test
			IITD	230	2601	
Deep learning-based methods	Izadpanahkakhk et al. [30]	FEM	IITD	230	2600	4 img/sub Train and remaining Test
			HKPU	193	7752	Images from first session Train and Images from second session Test
	Fei et al. [5]	AlexNet	IITD	230	2600	4 img/Sub randomly selected Train and remaining Test
		VGG-16	GPDS	100	1000	
		Inception-V3	CASIA	312	5500	
		ResNet-50				
	Zhao and Zhang [31]	DDR	IITD	230	2600	4 img/Sub randomly selected Train and remaining Test
			CASIA	312	5500	

**Table 2:** Identification accuracies using all BSIF parameters applied to the CASIA database.

	5 Bits	6 Bits	7 Bits	8 Bits	9 Bits	10 Bits	11 Bits	12 Bits
**3 × 3**	49.15	60.11	62.37	63.99	/	/	/	/
**5 × 5**	56.99	70.26	74.47	77.02	78.51	78.76	78.76	77.10
**7 × 7**	67.07	76.53	82.92	87.05	85.47	87.62	89.03	90.69
**9 × 9**	70.55	80.09	85.59	86.40	89.64	92.96	93.85	94.25
**11 × 11**	71.88	82.76	88.83	88.47	91.30	93.97	94.61	95.63
**13 × 13**	73.70	81.39	88.87	89.23	92.47	95.34	94.98	96.03
**15 × 15**	75.20	83.09	89.40	90.77	93.32	94.29	95.67	96.60
**17 × 17**	75.44	83.37	89.07	91.70	93.68	94.53	95.95	**96.35**

**Table 3:** Identification accuracies using all BSIF parameters applied to the IITD database.

	5 Bits	6 Bits	7 Bits	8 Bits	9 Bits	10 Bits	11 Bits	12 Bits
**3 × 3**	25.88	34.64	38.49	37.59	/	/	/	/
**5 × 5**	35.87	46.35	53.56	53.56	51.51	51.67	52.17	52.66
**7 × 7**	45.53	58.72	58.72	66.09	67.15	72.48	72.48	75.18
**9 × 9**	48.81	62.65	72.89	75.51	76.65	82.47	82.88	84.93
**11 × 11**	53.48	67.97	78.70	79.93	84.93	88.20	89.84	90.58
**13 × 13**	57.08	69.20	79.52	82.96	86.81	91.31	92.38	93.77
**15 × 15**	57.73	71.99	80.34	83.94	89.02	91.15	93.85	94.67
**17 × 17**	57.08	71.90	80.83	85.58	89.68	91.64	94.34	**95.49**

**Table 4:** Identification accuracies using several DWT families applied to the IITD database.

DWT Families	Haar	Daubechies		Biorthogonal		Coiflets		Symlets		Fejer-Korovkin Filters		Reverse Biorthogonal		Discrete Meyer
**Results**	96.23	db1	96.23	bior1.1	96.23	coif1	96.15	sym2	96.15	fk4	96.06	rbio1.1	96.23	96.15
		db2	96.15	bior2.2	96.23	coif2	**96.39**	sym3	96.06	fk6	96.15	rbio2.2	96.15	
		db3	96.06	bior3.1	96.15	coif3	96.15	sym4	96.15	fk8	96.31	rbio3.1	96.15	
		db4	**96.39**	bior3.9	96.31	coif4	**96.39**	sym5	96.15	fk14	96.31	rbio3.9	96.31	

**Table 5:** Identification accuracies using several DWT families applied to the CASIA database.

DWT Families	Haar	Daubechies		Biorthogonal		Coiflets		Symlets		Fejer-Korovkin Filters		Reverse Biorthogonal		Discrete Meyer
**Results**	96.92	db1	96.92	dior1.1	96.92	coif1	96.88	sym2	96.92	fk4	96.96	rbio1.1	96.92	96.19
		db2	96.92	dior2.2	96.92	coif2	97.04	sym3	96.88	fk6	96.92	rbio2.2	96.88	
		db3	96.88	dior3.1	96.96	coif3	97.08	sym4	96.92	fk8	97.00	rbio3.1	96.92	
		db4	97.04	dior3.9	97.04	coif4	97.08	sym5	97.04	fk14	97.00	rbio3.9	**97.12**	

**Table 6:** The performance of our system without and with pre-processing (median filtering or CLAHE) using the IITD and CASIA databases.

Databases	Without Pre-Processing (%)	Pre-Processing with Median Filtering (%)	Pre-Processing with CLAHE (%)
**IITD**	96.39	95.99	**97.13**
**CASIA**	97.12	96.60	**97.21**

**Table 7:** The performance of our system using K-NN vs. CDNN applied to IITD and CASIA databases.

	CASIA				IITD			
K	K-NN		CDNN		K-NN		CDNN	
	Euclidean	City Block	Euclidean	City Block	Euclidean	City Block	Euclidean	City Block
1	97.21	**98.10**	97.21	**98.10**	97.13	**98.77**	97.13	**98.77**
3	96.68	97.73	96.68	97.69	95.91	98.28	95.91	98.12
5	96.08	97.29	96.60	97.41	93.78	97.13	94.02	97.13
7	95.15	96.68	95.71	97.01	92.63	96.48	93.12	96.64
9	94.50	96.08	95.50	96.76	90.91	95.33	91.89	95.74

**Table 8:** Comparison of recognition rates with different recent approaches.

	Publication	Year	Method	IITD (%)	CASIA (%)
Coding-based methods	Kong and Zhang [20]	2004	CompCode	77.79	79.27
	Sun et al. [19]	2005	OrdinalCode	73.26	73.32
	Zhang et al. [21]	2012	E-BOCV	85.93	84.06
	Fei et al. [22]	2016	DOC	89.99	78.51
	Xu et al. [23]	2018	DRCC	88.82	/
Texture-based methods	Michael et al. [24]	2008	DGLBP	76.44	78.86
	Morales et al. [25]	2011	SIFT_OLOF	89.44	89.99
	Hammami et al. [26]	2014	LBP	/	96.66
	Wu et al. [27]	2014	SIFT_IRANSAC_OLOF	93.28	91.46
	Luo et al. [28]	2016	LLDP_MFRAT	92.75	90.77
			LLDP_Gabor	95.17	93.00
	Wu et al. [29]	2021	TFD	97.47	96.88
Deep learning-based methods	Izadpanahkakhk et al. [30]	2018	FEM	94.70	/
	Fei et al. [5]	2019	AlexNet	88.18	94.91
			VGG-16	92.12	94.01
			Inception-V3	96.22	93.85
			ResNet-50	95.57	95.21
	Zhao and Zhang [31]	2020	DDR	98.70	99.41
	**Our Approach**	**2022**	**BSIF + DWT**	**98.77**	**98.10**

## Data Availability

The employed data for the different experiments are taken from two public datasets and can be obtained from [18,19].
